# Mechanobiology: A New Frontier in Biology

**DOI:** 10.3390/biology10070570

**Published:** 2021-06-22

**Authors:** Tae-Jin Kim

**Affiliations:** 1 Department of Biological Sciences, Pusan National University, Pusan 46241, Korea; tjkim77@pusan.ac.kr; 2 Department of Integrated Biological Science, Pusan National University, Pusan 46241, Korea; 3 Institute of Systems Biology, Pusan National University, Pusan 46241, Korea

As we observe an increase in muscle mass by lifting weights or a significant mass loss in musculoskeletal tissues of astronauts returning after a stay in space, we note the manifestation of the mechanism of mechanotransduction that is central to mechanobiology. Observing the heart’s dynamic and long-standing regular activity from birth to death, we marvel at the heart’s ability to produce and utilize force. When listening to beautiful music, various sounds are converted into vibrations in our ears and interpreted in the brain, which also contains the elements of mechanobiology. Mechanobiology has grown naturally and enigmatically from biomechanics by integrating powerful elements of molecular and cellular biology. Therefore, while traditionally, the term “biomechanics” has dealt with the mechanical aspects of tissues or biological systems’ kinematic analysis, the modern term “mechanobiology” has become an interdisciplinary field that encompasses the mechanical cascade of biological events initiated or mediated by mechanical forces.

Mechanobiology is the study of how biological components such as cells, tissues, and organs can sense and respond to mechanical cues to regulate numerous biological processes, including development, differentiation, physiology, and diseases [1,2,3]. At the cellular level, the mechanical cues of the surrounding extracellular matrix (ECM) control normal cellular physiology. The processes of mechanosensing and mechanotransduction involve both intra- and extra-cellular components such as integrins, the ECM proteins, and the cytoskeleton (CSK). Integrins play a pivotal role in cellular mechanosensing by physically connecting the CSK to the ECM that forms clusters termed “focal adhesions (FAs)”. It is known that integrins recruit more than 150 proteins, including FA adapter proteins, shuttling proteins, and kinases, to the cell-ECM network [4,5]. Many FA proteins promote the Rho family of small GTPases such as RhoA, Rac, and CDC42 in mechanosensing [6]. The ECM is a complex protein meshwork that provides the scaffold for cell adhesion and mechanical support. The CSK consists of three types of protein filaments; actin, microtubules, and intermediate filaments that enable cells to maintain their shape and mechanical strength [7]. These elements eventually orchestrate various downstream signaling cascades to determine the fate and behavior of cells by regulating gene expression.

Although many biologists still regard mechanobiology as an emerging discipline, its origin dates back about 100 years. In 1917, biologist D’Arcy Thompson published a book titled “On Growth and Form”, in which he suggested that physical or mathematical principles could explain the structure and formation of patterns in plants and animals and that the shape of living organisms can reflect mechanical forces [8]. His work has provided the biology community with new and significant insights because evolution as the fundamental determinant of the structure and form of living organisms was mainstream at the time. It was the most emphasized field of biology overall, but the roles of physical laws and mechanics were underemphasized. Moreover, the research technologies at the time were inadequate to unveil Thompson’s questions, which remained unaddressed for many years.

Since the 1980s, many techniques, including mechanical measurement tools, fluorescent probes and imaging, and nano-micro manufacturing, have revolutionized research perspectives and scientific areas. In the 1980s, with the invention of the atomic force microscope (AFM) and optical tweezers, they quickly emerged as essential techniques that can be used for various applications in many sciences and engineering fields, including mechanobiology. The AFM has allowed us to apply and measure physical forces from the pN (piconewton) to the μN (micronewton) range on spatially defined areas, enabling the simultaneous morphological and mechanical characterization of complex biological systems [9]. The optical tweezers using a highly focused laser beam have been utilized to offer a repulsive or attractive force by manipulating small particles, which are now being used for mechanobiological studies such as mechanotransduction, cell micro-rheology, membrane mechanics, and cell–cell interactions [10]. In the 1990s, the invention of traction force microscopy, along with advances in nano- and micro-fabrication technologies, have allowed us to better understand the cellular and molecular mechanisms for how traction forces play a crucial role in many biological processes, including embryogenesis, inflammation, cancer metastasis, and angiogenesis [11,12]. Recently, the advent of super-resolution microscopy and single-molecule imaging techniques has expanded the molecular level of mechanobiology research by visually analyzing the dynamics of nucleic acids and proteins and various molecular interactions operated by mechanical forces [13,14]. In the future, novel technologies will emerge and offer more opportunities to unravel the underlying mechanism by which mechanical aspects govern biological systems.

Finally, the main goals of mechanobiology research can be threefold. Firstly, it aims to quantify or estimate the mechanical environments to which cells are exposed to health and disease. Secondly, it aims to identify and quantify the mechanical responses and the molecular mechanisms of pathological conditions caused by mechanical disturbance. Thirdly, it intends to ultimately apply the knowledge obtained to the development of new medical therapies. To achieve these three goals, mechanobiologists can be familiar with both the basic concepts of biomechanics and the modern means of cellular and molecular biology. This Special Issue will help shed light on advances in biology through interdisciplinary integration of physics, chemistry, mathematics, and engineering, focusing on the state-of-the-art knowledge of mechanobiology and the application of mechanobiology principles. We appreciate all contributors’ efforts in the creation of this Special Issue.
